# Fibrosing colonopathy associated with cysteamine bitartrate delayed-release capsules in cystinosis patients

**DOI:** 10.1007/s00467-024-06339-z

**Published:** 2024-03-11

**Authors:** Omayma A. Kishk, Ivone Kim, Carmen Cheng, Mukesh Summan, Monica A. Muñoz

**Affiliations:** 1 Division of Pharmacovigilance, Office of Surveillance and Epidemiology, Center for Drug Evaluation and Research, U.S. Food and Drug Administration, 10903 New Hampshire Ave, Silver Spring, MD 20993 USA; 2 Division of Pharmacology and Toxicology, Rare Diseases, Pediatrics, Urologic and Reproductive Medicine, Office of New Drugs, Center for Drug Evaluation and Research, U.S. Food and Drug Administration, 10903 New Hampshire Ave, Silver Spring, MD 20993 USA

**Keywords:** Cystinosis, Cysteamine delayed-release, Fibrosing colonopathy, Pharmacovigilance, Adverse event

## Abstract

**Background:**

The objective of this report is to identify and characterize cases of fibrosing colonopathy, a rare and underrecognized adverse event, associated with cysteamine delayed-release (DR) in patients with nephropathic cystinosis.

**Methods:**

We searched the U.S. Food and Drug Administration Adverse Event Reporting System (FAERS) and the medical literature for postmarketing reports of fibrosing colonopathy associated with cysteamine through August 2, 2023.

**Results:**

We identified four cases of fibrosing colonopathy reported with the use of cysteamine DR. The time to onset ranged from 12 to 31 months. In one case, the patient required surgery to have a resection of a section of the strictured colon and a diverting ileostomy. Fibrosing colonopathy was diagnosed by histopathology in two of the cases.

**Conclusions:**

Our case series identified the risk of fibrosing colonopathy in patients taking cysteamine DR and prompted regulatory action by the FDA. As outlined in changes to the U.S. prescribing information for cysteamine DR, healthcare professionals should be aware of the potential risk of fibrosing colonopathy with cysteamine DR, especially as symptoms can be non-specific leading to misdiagnosis or delayed diagnosis. If the diagnosis of fibrosing colonopathy is confirmed, consideration should be given to permanently discontinuing cysteamine DR and switching to cysteamine immediate-release treatment.

**Graphical abstract:**

**Figure:**
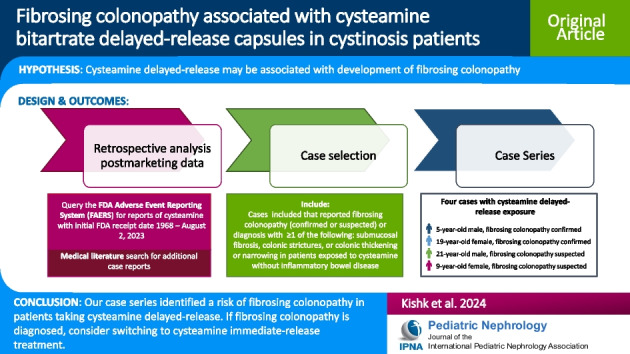
A higher resolution version of the Graphical abstract is available as Supplementary information

**Supplementary Information:**

The online version contains supplementary material available at 10.1007/s00467-024-06339-z.

## Introduction

Nephropathic cystinosis is an inherited lysosomal storage disorder caused by biallelic mutations in the *CTNS* gene resulting in the defective function of lysosomal cystine transporter (cystinosin) and the accumulation of cystine crystals in the kidneys and other organs affecting approximately 2000 individuals worldwide [1–3]. Management of nephropathic cystinosis consists of supportive treatment with replacement of renally lost compounds, nutritional support, hormonal replacement therapy, and cystine-depleting drug therapy [2].

In the U.S., Cystagon (cysteamine immediate-release (IR) capsules) was approved in 1994 [4], and Procysbi (cysteamine delayed-release (DR) capsules and granules) was approved in 2013 [5]. Both are indicated for the treatment of nephropathic cystinosis in patients 1 year of age and older. Cysteamine DR capsules and granules contain an equivalent amount of cysteamine-free base, formulated as the same microspheronized core beads with an enteric coating. The enteric coating on the beads consists of Eudragit L 30 D-55, triethyl citrate, and talc. The coating on the beads allows cysteamine DR beads to bypass gastric degradation of the active pharmaceutical ingredient (cysteamine), thus allowing for more convenient dosing from every 6 h with IR capsules to every 12 h with DR capsules and granules. Every 25 mg of cysteamine DR (capsules and granules) contains 18.92 mg of the inactive ingredient Eudragit L 30 D-55, a polymer of methacrylic acid copolymer (MAC) [5]. Other names for this polymer include Methacrylic Acid–Ethyl Acrylate Copolymer (1:1) Dispersion 30 Percent, Methacrylic Acid and Ethyl Acrylate Copolymer Dispersion, and Methacrylic Acid Copolymer LD. Cysteamine IR does not contain MAC [4].

Fibrosing colonopathy is characterized by colonic submucosal fibrosis due to the deposition of mature collagen leading to gradual stenosis of the colonic lumen. Histological findings include disruption of the muscularis mucosa and ulcerative inflammation that is distinct from findings of inflammatory bowel disease [6, 7]. Symptoms of fibrosing colonopathy include abdominal pain, abdominal distension, vomiting, constipation, bloody diarrhea, weight loss, and poor weight gain. Diagnosis of fibrosing colonopathy is based on clinical symptoms, diagnostic imaging studies, and histopathology. Treatment for fibrosing colonopathy includes oral steroids, colonic balloon dilatation, or colectomy [7]. Fibrosing colonopathy is a rare adverse event initially identified in patients with cystic fibrosis who received high doses of pancreatic enzyme replacement therapy containing MAC as an inactive ingredient. Leading hypotheses implicate MAC in the development of fibrosing colonopathy in patients with cystic fibrosis [8, 9]; however, the mechanism by which MAC may induce gastrointestinal toxicity is unknown. Prior to the evaluation of fibrosing colonopathy with the use of cysteamine DR, fibrosing colonopathy was included as an adverse event in the U.S. prescribing information for pancreatic enzymes. The purpose of this report is to identify and characterize cases of fibrosing colonopathy associated with cysteamine DR reported to the U.S. Food and Drug Administration (FDA) Adverse Event Reporting System (FAERS) and the medical literature in patients with nephropathic cystinosis.

## Methods

We conducted a retrospective analysis to identify postmarketing cases of fibrosing colonopathy reported with cysteamine. We searched the FAERS database for postmarketing reports of fibrosing colonopathy associated with cysteamine received by the FDA from 1968 (first reports in FAERS) through August 2, 2023. FAERS is a computerized repository of spontaneous adverse event reports submitted by product manufacturers, consumers, and healthcare professionals from U.S. and non-U.S. sites [10]. The database is designed to support FDA’s postmarketing safety surveillance program for drug and therapeutic biologic products. Adverse events are coded using Medical Dictionary for Regulatory Activities (MedDRA) terminology [11].

To identify cases of fibrosing colonopathy, we searched the FAERS database using the following MedDRA-Preferred Terms: Fibrosing colonopathy, Intestinal fibrosis, Fibrosis, Large intestinal stenosis, Intestinal stenosis, Gastrointestinal stenosis, Stenosis, Intestinal strangulation, Gastrointestinal wall thickening, Intestinal mucosal hypertrophy, Distal intestinal obstruction syndrome, Intestinal obstruction, Large intestinal obstruction, Gastrointestinal obstruction, Large intestinal obstruction reduction, Intestinal pseudo-obstruction, Obstruction, Functional gastrointestinal disorder, and Intestinal dilation. We searched PubMed and Embase for publications through August 2, 2023, to identify any additional case reports in the medical literature.

We included cases that described patients without a history of inflammatory bowel disease who were exposed to cysteamine and reported either (1) a confirmed or suspected diagnosis of fibrosing colonopathy or (2) at least one of the following findings: submucosal fibrosis, colonic strictures, or colonic thickening or narrowing.

## Results

We identified four FAERS cases that met our inclusion criteria, including one case that was also published in the medical literature [12]. All cases reported the use of cysteamine DR capsules either describing fibrosing colonopathy or manifestations of fibrosing colonopathy. Two of the four cases had a definitive diagnosis of fibrosing colonopathy by histopathology. In the remaining two cases, fibrosing colonopathy was suspected but not confirmed. Table 1 displays the case characteristics. The median age for patients in our case series was 14 years (range 5–21 years). In two cases that provided information for dose evaluation, the cysteamine DR dosing did not approximate maximum daily doses per the product labeling. Reported symptoms included vomiting, abdominal pain, bloody or persistent diarrhea, and fecal incontinence. In one case, latency to symptoms was 26 months, and latency to fibrosing colonopathy diagnosis was 5 months after symptom development. Another case reported that the onset of symptoms was approximately 12 months after initiation of cysteamine DR. The remaining two cases did not report a time to onset of symptoms or time of diagnosis. Cysteamine DR was changed to cysteamine IR in one case. Cases did not report on long-term outcomes such as reoccurrence of fibrosing colonopathy symptoms.

**Table 1:** Summary of postmarketing fibrosing colonopathy cases associated with cysteamine DR (*n* = 4)

	Case 1 [12]	Case 2	Case 3	Case 4
Initial FDA received (year)	2021	2017	2019	2019
Country of origin	USA	Norway	Norway	Austria
Age (y)/sex	5/male	21/male	19/female	9/female
Weight (kg)	20	Not reported	Not reported	25
Daily dose of cysteamine DR at time of event	1050 mg (525 mg BID)	1350 mg (675 mg BID)	Not reported	1200 mg (600 mg BID)
Time to event onset after cysteamine DR initiation	31 months	12 months	Not reported	Not reported
Concomitant medications	Famotidine, potassium phosphate and sodium phosphate, potassium chloride, cholecalciferol, ondansetron, loperamide, levocarnitine, levothyroxine	Cysteamine ophthalmic drops, enalapril, tacrolimus, levothyroxine, mycophenolate mofetil	Methacrylic acid–ethyl acrylate copolymer (1:1)^a^	Not reported
Symptoms of suspected fibrosing colonopathy	Bloody diarrhea, worsening vomiting, abdominal pain	Fecal incontinence, diarrhea	Not reported	Not reported
Definitive diagnosis of fibrosing colonopathy	Yes, by pathology	No, suspected; patient referred to gastroenterologist for “examination and exclusion of fibrosing colonopathy”	Yes, by histology	No, but colonic strictures identified
Disposition of cysteamine DR	Discontinued switched to cysteamine IR	Unknown	Not reported	Continued
Fibrosing colonopathy outcome	Resolving	Unknown	Unknown	Resolved
Case summary	A physician reported that a 5-year-old male experienced bloody diarrhea, worsening vomiting, and abdominal pain about 26 months after starting cysteamine DR. Five months later, he underwent a colonoscopy. The procedure was complicated by perforation of a colonic stricture with subsequent resection of the perforated bowel. Pathology report for the resected sample was consistent with fibrosing colonopathy	A physician reported that a 21-year-old male experienced partial fecal incontinence and diarrhea approximately 12 months after starting cysteamine DR. The patient was referred to a gastroenterologist for “examination and exclusion of fibrosing colonopathy”	A physician reported that a 19-year-old female experienced fibrosing colonopathy, confirmed by histology	A physician reported that a 9-year-old female experienced colonic stricture after an unknown duration on cysteamine DR. It was reported that the event resolved after the patient removed cysteamine DR beads from the cysteamine DR hard gelatin capsules and transferred them into soft gelatin capsules

*BID* twice daily, *DR* delayed-release, *IR* immediate-release

^a^As reported in case narrative

## Conclusions

Our evaluation of cysteamine DR and fibrosing colonopathy did not identify specific risk factors for the occurrence of fibrosing colonopathy such as dose, age, duration of use, or formulation as there is limited data to make such determinations. Although a singular report of fibrosing colonopathy with cysteamine DR has been reported in the literature [12], this is the first case series reported involving cysteamine DR and fibrosing colonopathy.

Fibrosing colonopathy is a rare adverse event and may present with non-specific abdominal symptoms. Patients in our case series with suspected or confirmed fibrosing colonopathy presented with abdominal pain, vomiting, bloody or persistent diarrhea, and fecal incontinence. This suggests that patients treated with cysteamine DR who develop severe, persistent, and/or worsening abdominal symptoms may need evaluation for fibrosing colonopathy.

Cysteamine DR dosing is titrated based on cystine levels, which could result in varied MAC exposure. In two cases that reported weight information and cysteamine DR dosage, the cysteamine DR dosage was consistent with the recommended maintenance dosage. Based on the extrapolated body surface area for these cases, the dosage did not exceed the maximum daily cysteamine DR dosage of 1.95 g/m^2^. Currently, there is no evidence in the published literature that higher doses of cysteamine DR are associated with a higher risk of fibrosing colonopathy. Nonclinical studies hypothesize that colonic exposure to drugs is generally higher in children when compared to adults due to a decreased ratio of colonic surface area to body weight [13, 14]. Within this framework, cysteamine DR colonic exposure in pediatric patients may be higher than that of adults since cysteamine DR is dosed by weight and body surface area. By extension, colonic exposure levels of MAC in children could be as much as 10 times higher than in adults [15]. Published studies provide insufficient detail to definitively establish an association between MAC and the occurrence of fibrosing colonopathy [14–17]. Although pediatric patients with lower weights may have higher exposure to MAC, there is limited nonclinical evidence supporting an increased risk of fibrosing colonopathy. However, it appears that there are sufficient safety margins of exposure in animals to the maximum MAC daily content in cysteamine DR based on the completed adult animal studies in which no overt signs of clinically significant gastrointestinal pathology were observed [18].

It is not known if there is a link between chronicity of cysteamine DR use and fibrosing colonopathy. Two patients in the case series received cysteamine DR for 12 to 31 months prior to the suspected fibrosing colonopathy diagnosis. In patients with cystic fibrosis, the development of fibrosing colonopathy occurred 12 to 24 months after “high-dose” pancreatic enzyme replacement therapy use [13]. Without further studies, it is difficult to determine whether cysteamine DR’s active or inactive components contribute to the development of fibrosing colonopathy. However, considering the available information in its entirety, we hypothesize that MAC may play a role in the development of fibrosing colonopathy. MAC is found on the enteric coating of cysteamine DR products’ beads and not on the outer capsule. In one case, although the patient transferred the cysteamine DR beads from one capsule to another, the patient was still exposed to MAC. While this case described the resolution of colonic strictures despite continued use of cysteamine DR at the time of reporting, it did not specify if the patient received treatment or if the cysteamine DR dose was decreased after the occurrence of the strictures. Our FAERS search did not identify any cases of fibrosing colonopathy occurring in patients on cysteamine IR despite it being FDA-approved 15 years prior to the cysteamine DR formulation. The lack of data may also be attributed to the rarity of fibrosing colonopathy [7] and cystinosis [1].

FAERS has many strengths, such as the identification of rare adverse events; however, our evaluation has some limitations. Reporting to FAERS is not predicated on establishing a causal relationship between a product and an event, and reports do not always contain enough information to properly evaluate an event. Additionally, limited information about nephropathic cystinosis also affects our interpretation of findings as it is unknown whether patients with nephropathic cystinosis are inherently at high risk for developing fibrosing colonopathy as compared with the general population. While it is not possible to calculate incidence rates using FAERS data, we note that reporting fibrosing colonopathy as a suspected adverse reaction to FAERS is extremely rare. Of the > 25 million reports in FAERS as of December 31, 2023, fewer than 30 include fibrosing colonopathy as an adverse event. Half of these are associated with pancreatic enzyme replacement therapies. Consequently, considering the rarity of the event in conjunction with the utilization of a product for a rare disease, we decided to include cases without confirmatory diagnostic information and other details that would support a more rigorous assessment. Despite these limitations, our evaluation provides compelling support for the hypothesis of a relationship between fibrosing colonopathy and cysteamine DR formulation.

Our case series identified the risk of fibrosing colonopathy in patients taking cysteamine DR and prompted regulatory action by the FDA [5]. In February 2022, the FDA updated the cysteamine DR prescribing information with a warning for fibrosing colonopathy. Prescribers and other health care professionals caring for patients with nephropathic cystinosis should be aware of the potential risk of fibrosing colonopathy with cysteamine DR, as symptoms can be non-specific leading to misdiagnosis or delayed diagnosis. It is important to convey this risk to patients and switch to cysteamine IR if fibrosing colonopathy is confirmed.

### Supplementary Information

Below is the link to the electronic supplementary material.

Graphical abstract (PPTX 86 KB)

## Data Availability

Data archiving is not mandated, but data will be made available on reasonable request. FAERS data are available via the FAERS Public Dashboard and as Quarterly Data files. Additionally, individual case reports can be requested via a Freedom of Information Act request to the FDA. Additional details can be found at https://www.fda.gov/drugs/surveillance/questions-and-answers-fdas-adverse-event-reporting-system-faers.

## Abbreviations

IR: Immediate-release
DR: Delayed-release
FDA: Food and Drug Administration
FAERS: FDA Adverse Event Reporting System
MAC: Methacrylic acid copolymer
MedDRA: Medical Dictionary for Regulatory Activities

## Declarations

### Disclaimer

This article reflects the views of the authors and should not necessarily be construed to represent Food and Drug Administration’s views or policies.
