# Supplementary material for: Fibrosing colonopathy associated with cysteamine bitartrate delayed-release capsules in cystinosis patients
Source: Pediatr Nephrol. 2024 Mar 11;39(8):2429–33. doi: 10.1007/s00467-024-06339-z (PMC11199274; doi:10.1007/s00467-024-06339-z)
Supplement: Supplementary file 1 — Graphical abstract (PPTX 86 KB) [file 467_2024_6339_MOESM1_ESM.pptx]

## Slide 1
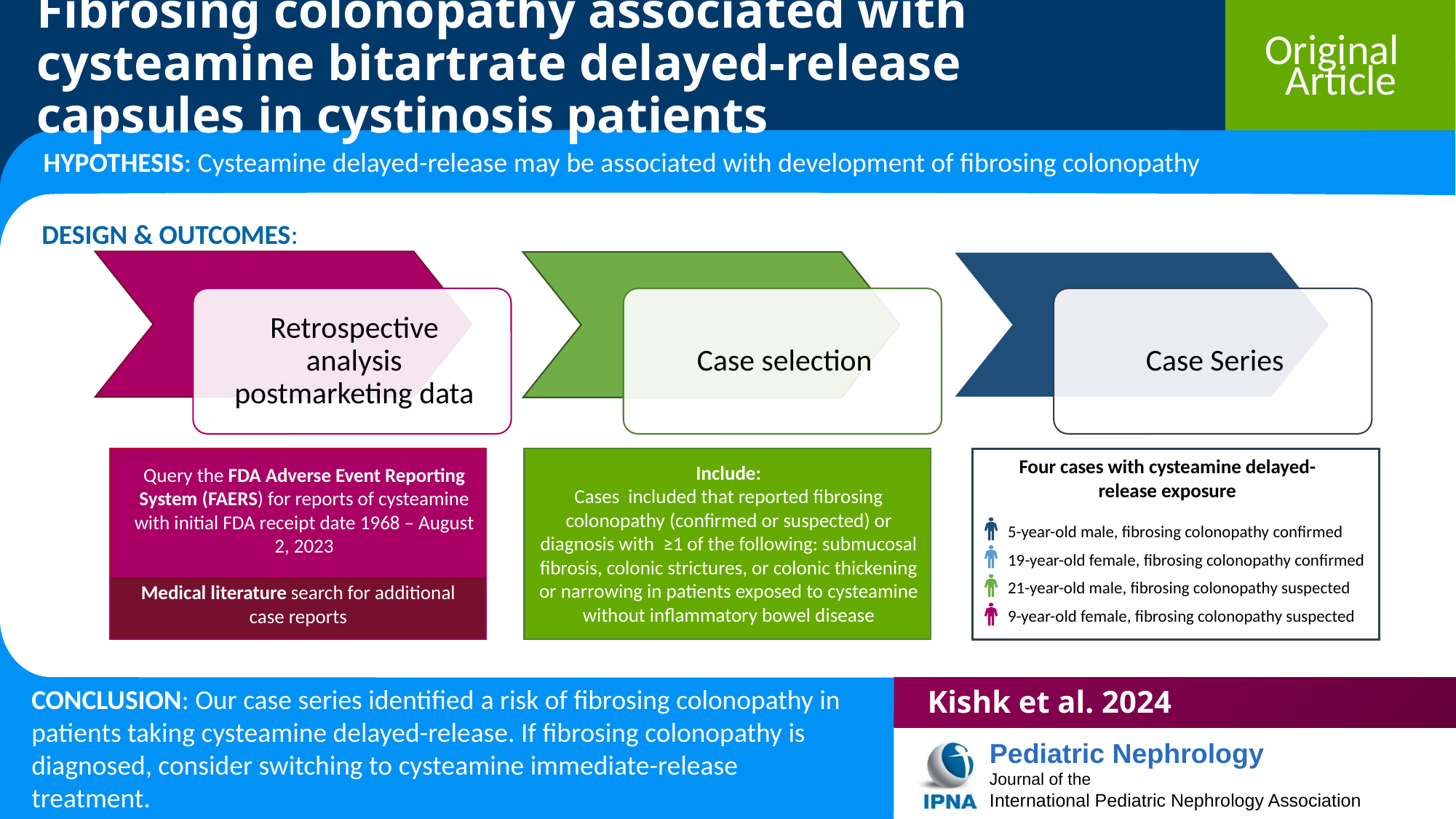

Fibrosing colonopathy associated with cysteamine bitartrate delayed-release capsules in cystinosis patients
HYPOTHESIS: Cysteamine delayed-release may be associated with development of fibrosing colonopathy
DESIGN & OUTCOMES:
Query the FDA Adverse Event Reporting System (FAERS) for reports of cysteamine with initial FDA receipt date 1968 – August 2, 2023
Medical literature search for additional case reports
Four cases with cysteamine delayed-release exposure
5-year-old male, fibrosing colonopathy confirmed
19-year-old female, fibrosing colonopathy confirmed
21-year-old male, fibrosing colonopathy suspected
9-year-old female, fibrosing colonopathy suspected
Include:
Cases included that reported fibrosing colonopathy (confirmed or suspected) or diagnosis with ≥1 of the following: submucosal fibrosis, colonic strictures, or colonic thickening or narrowing in patients exposed to cysteamine without inflammatory bowel disease
CONCLUSION: Our case series identified a risk of fibrosing colonopathy in patients taking cysteamine delayed-release. If fibrosing colonopathy is diagnosed, consider switching to cysteamine immediate-release treatment.
Kishk et al. 2024
